# Nonvesicular trafficking of cholesterol by Aster proteins

**DOI:** 10.1093/lifemeta/load003

**Published:** 2023-01-18

**Authors:** Dougall Norris, Yvette Aw, Hongyuan Yang

**Affiliations:** School of Biotechnology and Biomolecular Sciences, University of New South Wales, Sydney, NSW, 2052, Australia; School of Biotechnology and Biomolecular Sciences, University of New South Wales, Sydney, NSW, 2052, Australia; School of Biotechnology and Biomolecular Sciences, University of New South Wales, Sydney, NSW, 2052, Australia

**In a recent article published in *Nature Metabolism*, Peter Tontonoz and colleagues found that the Aster/GramD1 proteins were required for plasma membrane (PM) cholesterol to reach the endoplasmic reticulum (ER) in mouse liver during fasting, low-density lipoprotein (LDL) uptake, or reverse cholesterol transport (RCT). The Aster/GramD1 pathway plays a key role in maintaining hepatic and systemic cholesterol/lipid homeostasis.**

Cholesterol is an essential constituent of organellar membranes in mammalian cells [1]. Although cholesterol is synthesized in the endoplasmic reticulum (ER), the level of cholesterol in the ER is very low: ER cholesterol accounts for ~1% of total cellular free cholesterol and <5% of total ER lipids [2]. Instead, up to 90% of cellular free cholesterol exists in the plasma membrane (PM) where it makes up 40%−50% of PM lipids and plays a critical role in maintaining the stability and function of the PM [3, 4].

The level of PM cholesterol is tightly regulated primarily by the ER localized cholesterol sensing machinery: sterol-regulatory element binding protein (SREBP) cleavage-activating protein (SCAP) and insulin-induced gene (INSIG) [5]. This is facilitated by the low-cholesterol content of the ER, which enables SCAP-INSIG to sense small perturbations of cholesterol in the ER to activate/inhibit SREBP-2, a key transcription factor governing cholesterol synthesis and uptake [6]. Thus, the small pool of cholesterol in the ER can monitor cholesterol concentration in the large PM pool. How is this achieved? Recent elegant studies provided mechanistic insights into this regulation. Das *et al*. demonstrated that there are three distinct pools of cholesterol in the PM: an essential pool (~12% of PM lipids) that is sequestered presumably by phospholipids and proteins; a sphingomyelin (SM)-sequestered pool (~15% of PM lipids) that can be released only when SM is hydrolyzed by sphingomyelinases (SMases); and a labile/expandable pool (~1%−16% of PM lipids depending on availability of exogenous cholesterol) that is freely accessible to cholesterol carriers for transport to the ER and possibly other organelles [3]. Under normal conditions, only the labile pool of PM cholesterol can be detected by domain 4 of anthrolysin O (ALOD4), a specific cholesterol probe [3]. The Aster family of proteins (Aster-A, -B, -C, encoded by Gramd1-a, Gramd1-b, Gramd1-c, respectively) were recently discovered to transfer accessible PM cholesterol to the ER [7]. Each Aster protein contains three key domains: an N-terminal GRAM domain that senses and binds to PM cholesterol and phosphatidylserine, a StART-like domain that transfers cholesterol, and a C-terminal transmembrane domain that anchors the protein to the ER. In the steady state, Aster proteins localize to the ER. When the level of PM cholesterol is increased, Aster proteins are recruited to ER-PM contact sites through the GRAM domain to enable the transfer of PM cholesterol to the ER by the StART-like domain, thereby increasing ER free cholesterol and suppressing SREBP-2. Conversely, loss of Aster function in cells expands the accessible PM cholesterol pool and reduces ER cholesterol, triggering the activation of the SREBP-2 pathway. In Aster-B deficient adrenal glands, the movement of high-density lipoprotein (HDL)-derived cholesterol from the PM to the ER is nearly completely blocked [7]. Together, these findings suggest that the labile/accessible pool of PM cholesterol can communicate rapidly with the ER cholesterol pool through the action of Aster proteins, enabling the ER cholesterol pool to monitor and control the level of PM cholesterol.

Much of the current information on Aster function and the partition of PM cholesterol into three distinct pools was obtained from cell line-based studies. It is necessary to investigate the physiological relevance of Aster-mediated cholesterol transfer in animals, especially in the liver, a key organ in systemic metabolism. A new study in *Nature Metabolism* [8] provided the first in-depth analysis of Aster function in the liver under physiological contexts. The authors hypothesized that Aster-mediated cholesterol transfer may be required during fasting and reverse cholesterol transport (RCT). Fasting is known to suppress hepatic cholesterol biosynthesis but not cholesteryl ester (CE) synthesis and bile acid production. In fact, CE synthesis is increased during fasting, despite reduced cholesterol biosynthesis. The source of cholesterol that sustains CE synthesis and bile acid production during fasting is unknown. Aster-A and -C but not -B are highly expressed in the liver. To determine the role of Aster in hepatic lipid metabolism, the authors generated L-A/C knockout (KO) mice that are deficient in both Aster-A and -C. Cholesterol accumulated on the surface of primary hepatocytes derived from the L-A/C KO mice, consistent with the results from cell line studies. L-A/C KO mice also showed upregulation of SREBP-2 target genes, suggesting lack of ER cholesterol. Importantly, prolonged fasting (16 h) reduced PM cholesterol in the liver of control mice, but not L-A/C KO mice. Moreover, fasting-induced CE accumulation in the liver was slightly reduced in the L-A/C KO mice, and became significantly reduced when mice were fed a statin diet to inhibit the compensatory increase in cholesterol biosynthesis. Together, these data indicate that during fasting, Aster-A and -C likely mediate the transfer of PM cholesterol to the ER, where cholesterol is converted to CE. In the absence of Aster-A and -C, fasting-induced CE synthesis was reduced.

Next, the authors determined the source of cholesterol for increased CE synthesis during fasting. Prolonged fasting reduced SM abundance in the liver, which was triggered by unsaturated fatty acids (such as arachidonic acid or oleic acid) released from adipose tissue. Oleate treatment promoted the hydrolysis of SM and increased the accessible pool of cholesterol in hepatocytes, and this increase was blocked when an inhibitor of neutral SMases was added. Thus, it appears that by promoting SM hydrolysis, adipocyte-derived fatty acids released by fasting can liberate the pool of cholesterol normally sequestered by SM. The authors further identified *Smpd3,* one of three neutral SMases in the liver, as the key enzyme linking fasting with increased hepatic cholesterol. The mRNA and protein levels of Smpd3 were increased in the mouse liver after a 16-h fast, and liver-specific overexpression of Smpd3 decreased hepatic SM levels, expanded the accessible/labile pool of PM cholesterol, and promoted cholesterol movement to the ER. Aster A and C carry the cholesterol released by Smpd3-mediated SM hydrolysis during fasting to the ER for CE synthesis to support the production of very low-density lipoprotein (VLDL): L-A/C KO mice had impaired liver VLDL secretion and reduced plasma apoB 100 levels. Overall, the first part of the manuscript explained how fasting can increase hepatic CE formation and unveiled a critical role of Aster-A/C in this pathway. Exactly how fasting/oleate increases the expression and activity of Smpd3 was not addressed in this study, but will be an interesting topic for future investigations. Moreover, it would be interesting to examine the expression of all putative cholesterol transporters in the L-A/C hepatocytes since the transfer of PM cholesterol to the ER was significantly but not completely blocked in these cells.

The authors then investigated the role of Aster -A/-C in the metabolism of lipoprotein-derived cholesterol. LDL particles bind LDL receptors to enter hepatocytes in a process called receptor-mediated endocytosis. LDL-derived cholesterol, once released from lysosomes via Niemann Pick C1/2 proteins, first reaches the PM before moving to the ER. Aster proteins were shown to deliver LDL cholesterol from the PM to the ER in cultured cell lines, but whether this is the case *in vivo* is not clear. The authors intravenously injected [^14^C] cholesterol-labeled LDL to WT and L-A/C KO mice, and found that [^14^C]-labeled CE accumulation was markedly lower in the livers of L-A/C KO mice, suggesting impaired delivery of LDL cholesterol to the ER. Consistently, SREBP-2 target genes were higher in the livers of L-A/C KO mice. Thus, Aster proteins can deliver LDL cholesterol to the ER *in vivo*.

Distinct from LDL, HDL delivers cholesterol to hepatocytes in the form of CE via selective uptake by scavenger receptor Class B Type 1 (SR-B1, an HDL receptor). Subsequently, HDL-derived CE is hydrolyzed by a yet-to-be identified esterase. HDL cholesterol is then believed to enter the accessible pool of PM cholesterol before reaching the ER for CE synthesis and bile acid formation. Indeed, HDL loading of cells for 1 h increased the PM labile cholesterol pool as indicated by higher ALOD4 staining. As with LDL loading, accumulation of CE after HDL loading was also lower in the livers of L-A/C KO mice, indicating a role for Aster A/C in delivering HDL cholesterol to the ER. HDL-derived cholesterol is also a known precursor for bile acid synthesis, and L-A/C KO mice had lower incorporation of cholesterol into fecal bile acids. These data further demonstrate that Aster-mediated cholesterol transfer promotes hepatic HDL cholesterol metabolism and is required for RCT. As the liver is a key organ in systemic lipid homeostasis, the authors investigated if Aster-mediated hepatic cholesterol trafficking is required for whole body lipid homeostasis. Total plasma cholesterol and especially plasma HDL were both higher in L-A/C KO mice. As trafficking to the ER was blocked under Aster A/C deficiency, PM cholesterol of hepatocytes was instead effused to ApoA1/HDL and stored in peripheral tissues including the adrenal glands. Despite higher plasma cholesterol and HDL levels in L-A/C KO mice, whole body cholesterol content was similar, suggesting that HDL efflux and incorporation into peripheral tissue compensate for cellular-trafficking defects. This work reveals a finely tuned homeostasis between hepatic intracellular cholesterol transport and systemic cholesterol metabolism.

In summary, results by Xiao *et al*. provide convincing evidence that the nonvesicular transfer of cholesterol from the PM to the ER by Aster proteins contributes significantly to hepatic and systemic lipid homeostasis. Results here also challenge the conventional wisdom that multiple redundant pathways must exist to mediate the movement of cellular lipids. Clearly, transfer of PM cholesterol to the ER is primarily mediated by Aster proteins in a variety of cell lines and tissues. Finally, the nonvesicular trafficking of lipids has always been a very difficult subject to study due to lack of live trackers of lipid movement. Much of the effort in this important area has been limited to *in vitro* work using cell lines. The current study by Xiao *et al*. has taken the investigation of lipid trafficking to physiological contexts and animal studies, and may therefore serve as a guide for future studies on the physiological significance of other nonvesicular lipid transfer pathways.
